# Direct Revascularization With the Angiosome Concept for Lower Limb Ischemia: A Systematic Review and Meta-Analysis

**DOI:** 10.1097/MD.0000000000001427

**Published:** 2015-08-28

**Authors:** Tzu-Yen Huang, Ting-Shuo Huang, Yao-Chang Wang, Pin-Fu Huang, Hsiu-Chin Yu, Chi-Hsiao Yeh

**Affiliations:** From the Department of Thoracic and Cardiovascular Surgery (T-YH, Y-CW, P-FH, C-HY), Chang Gung Memorial, Hospital, Keelung; College of Medicine (T-YH, T-SH, C-HY), Chang Gung University, Tao-Yuan; Department of General Surgery (T-SH), Chang Gung Memorial Hospital, Keelung; and Department of Nursing (H-CY), Chang Gung Memorial Hospital, Keelung, Taiwan, ROC.

## Abstract

The angiosome concept provides practical information regarding the vascular anatomy of reconstructive and vascular surgery for the treatment of peripheral arterial occlusive disease and, particularly, critical lower limb ischemia.

The aim of the study was to confirm the efficacy of direct revascularization with the angiosome concept (DR) for lower limb ischemia.

Complementary manual searches were performed through the Pubmed, Cochrane Library, and EMBASE databases.

We searched all randomized and nonrandomized studies (NRSs) comparing DR with indirect revascularization (IR) (without the angiosome concept) for lower limb ischemia. Only 9 nonrandomized controlled retrospective cohort studies were found and included. Trials published in any language were included.

Primary endpoints were time to limb amputation and time to wound healing. Data extraction and trial quality assessment were performed by two authors independently. A third author was consulted for disagreements settlement and quality assurance.

Five NRSs involving 779 lower limbs revealed that DR significantly improved the overall survival of limbs (hazard ratio [HR] 0.61; 95% confidence interval [CI] = 0.46–0.80; *P* < 0.001; *I*^2^ = 0%). In addition, DR significantly improved time to wound healing (HR 1.38; 95% CI = 1.13–1.69; *P* = 0.002; *I*^2^ = 0%, in 5 studies including 605 limbs).

All included studies were retrospective comparative studies, and no consensus was obtained in describing wound conditions in the included studies.

Our results suggested that treatment of lower limb ischemia using DR is more effective in salvaging limbs and healing wounds than IR is. Additional randomized controlled studies are necessary to confirm these results.

## Abstract 2

Supplemental Digital Content is available in the text

## INTRODUCTION

Peripheral arterial occlusive disease (PAOD) is a major disease that limits active aging in elderly people. Complications of PAOD are the leading cause of hospitalization and amputation for people with lower limb ischemia, and account for billion-dollar expenditures annually in the United States.^1^ Treatment goals for lower legs critical limb ischemia (CLI) patients are to increase wound healing, improve quality of life, prevent limb loss, and prolong survival. Guidelines from the Transatlantic Inter-Society Consensus II (TASC-II) and the American College of Cardiology/American Heart Association recommend multidisciplinary approaches to reduce the frequency of foot complications in CLI patients.^2,3^ Early revascularization intervention with bypass or endovascular surgery, particularly for high-risk patients, is considered to be the gold standard in reducing the possibility of hospitalization and amputation.^2,4–6^ Nevertheless, current revascularization intervention strategies, which restore circulation to a nontargeted artery, have a 15% failure rate in healing CLI wounds. Such a high rate suggests that increasing adequate blood supply to feeding arteries at the distal occluded lesion-site might be crucial in improving the results of intervention.

The concept of angiosomes, first described by Ian Taylor, provides practical information on the application of vascular anatomy for reconstruction and vascular surgery in the treatment of PAOD, and particularly on the treatment of CLI.^7,8^ According to the angiosome concept, the foot is divided into 6 distinct angiosomes fed by source arteries, 3 from posterior tibial, 2 from peroneal, and 1 from anterior tibial artery, with functional vascular interconnections between muscle, fascia, and skin.^9^ Numerous direct arterial-to-arterial connections exist between the main arteries of the foot, and these connections provide alternative routes of blood flow when the arteries that directly supply the angiosome is either disrupted or compromised.^9^ Therefore, the angiosome concept suggests that recanalization of the artery that is directly supplying the ischemic and/or ulcerated angiosome, instead of revascularizing one of the other 2 major arteries hoping that existing arterial-to-arterial connections will provide blood perfusion to the ischemic and/or ulcerated angiosome, might be more successful.^2,9^

It is unclear whether direct revascularization with the angiosome concept (DR) can provide superior results for CLI patients than that of conventional indirect revascularization (IR) without the angiosome concept. One recent review revealed that evidence is insufficient to recommend DR in CLI patients.^10^ However, we performed a systematic review and meta-analysis investigating the efficacy of DR, comparing it with conventional IR for the treatment of CLI patients.

## METHODS

### Search Strategy

This protocol-driven and systematic review was conducted in accordance with PROSPERO published protocol and analysis planning (PROSPERO 2013:CRD42013004401, http://www.crd.york.ac.uk/Prospero/).^11^ Searches were not restricted by publication status, date, or language. The search keywords included *angiosome*, *angioplasty*, *endovascular*, *revascularization*, *endoluminal*, *transluminal*, and *bypass*. In addition, MeSH terms were explored. The final results were combined with the following keywords: *lower limbs*, *extremities*, and *foot*. The databases we used to conduct our searches were Pubmed (from 1948 to March 2013), the Cochrane Library (latest issue published March 2013), and EMBASE (January 1980–March 2013). The search strategy is provided in Appendix 1. Databases of clinical trials (available at http://www.clinicaltrials.gov [accessed March 26, 2014]) reference lists of reviews had also been searched to identifying relevant trials.

### Study Selection

In the literature search, titles, abstracts, and full texts of trials identified were independently screened by three authors (T-YH, T-SH, and C-HY). Articles with comparisons between DR and IR were included if adults (≥18 years old) with critical lower limb ischemia (as defined by TASC-II) had been treated using either endovascular surgery or conventional bypass surgery.^12^ Reviews, case series, case reports, and trials without comparisons between DR and IR were excluded. Primary outcomes included time to limb amputation, time to wound healing, and mortality rate.

### Data Extraction

Characteristics of studies (year of publication, study design and setting, method of recruitment, inclusion and exclusion criteria), participants (sex, age, and underlying disease), interventions (operative techniques, endovascular, or traditional surgery), comparisons (types of control group), and outcomes (various outcome measurements and follow-up times) were recorded. In studies with multiple arm designs, head-to-head comparison data were extracted for data synthesis. Time to amputation and time to wound healing were used as the primary outcomes.

### Assessment of Risk of Bias

Data extraction and quality assessment were performed by two authors (T-YH and C-HY) independently. A third author (T-SH) was consulted for disagreements settlement and quality assurance. The risk of bias tool of the Systematic Reviews of Interventions from the Cochrane Handbook was utilized for determination of methodological quality.^12^ Because the included studies were all nonrandomized studies (NRSs), we assessed methodological quality by using the Newcastle–Ottawa Quality Assessment Scale (NOS).^13^ Three determinants composed the NOS system, including selection scores, outcome scores, and comparability scores.^13^ Studies with NOS ≥8 points were defined as high-quality studies, whereas other studies as low-quality studies.

### Data Synthesis and Statistical Analysis

For time to event outcome, hazard ratios (HRs) with 95% confidence intervals (CIs) were extracted in primary studies as the size to estimate the overall effect of treatment. If data were not provided, the effect size was calculated in accordance with methods suggested by Parmar et al^14^ by using a spreadsheet developed by Tierney et al,^15^ as described earlier. For 12-month amputation rate and 12-month wound healing rate, we defined amputation and wound healing as events. Clinical heterogeneity were assessed by comparing the protocols and methodologies of the included studies, and assessed statistical heterogeneity with the χ^2^ test results (using a cutoff value of *P* < 0.10), and the *I*^2^ statistic, where *I*^2^ < 25%, 25% ≤ *I*^2^ ≤ 50%, and *I*^2^ > 50% indicates mild, moderate, and substantial heterogeneity, respectively.^16,17^ Subgroup analysis based on study quality or intervention (surgical or endovascular) was conducted, and data synthesis and statistical analysis were conducted using Review Manager (RevMan Version 5.2; The Nordic Cochrane Center, Copenhagen, Denmark). A funnel plot was created to evaluate publication bias, and significance level was set at 0.05.

## RESULTS

### Search Results and Study Characteristics

**FIGURE 1:**
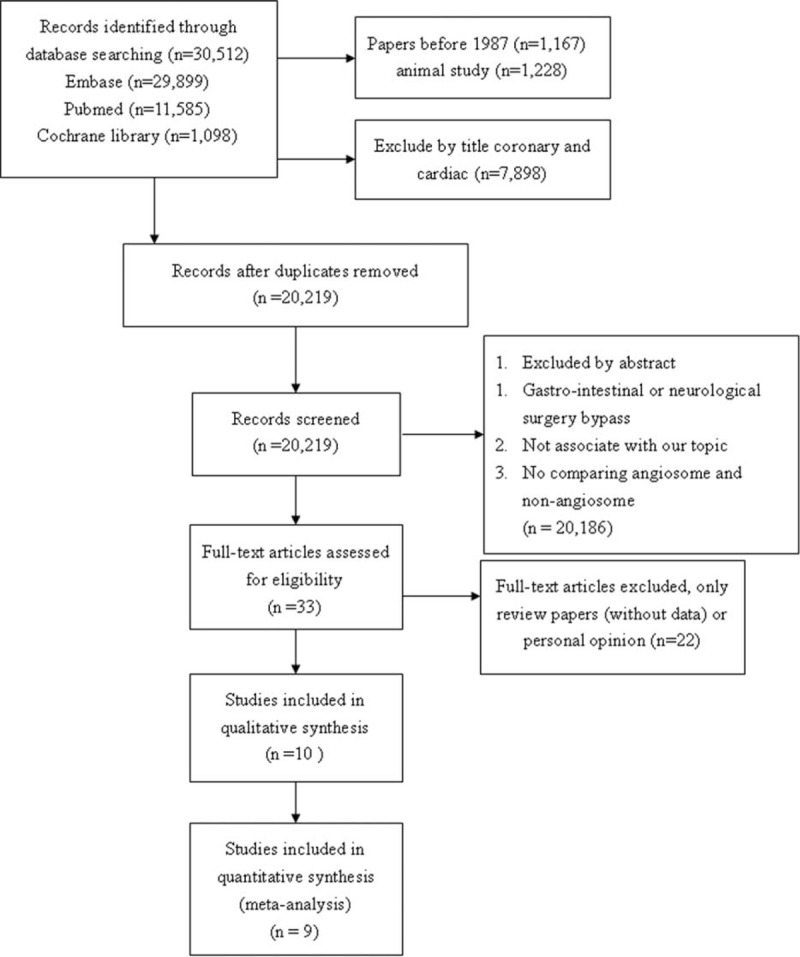
Flow diagram of the article selection process in accordance with PRISMA guideline. PRISMA = Preferred Reporting Items for Systematic Reviews and Meta-Analyses.

Overall, 10 NRSs were included, as shown in Figure 1 and Table 1 .^18–27^ No randomized control trial was identified, and all studies were retrospective cohort study. Iida et al published two studies in 2010^20^ and 2012.^24^ The study of 2010 analyzed patients from April 2003 to August 2008. The study of 2012 analyzed patients from April 2004 to October 2010 using matching method. Therefore, we used the study of 2012 to conduct meta-analysis. All the DR studies followed the Taylor's angiosome concept. Three studies (Azuma et al, Iida et al, and Söderström et al)^23–25^, which utilized propensity score matched comparison between DR and IR groups, were analyzed. Table 1  shows the major characteristics of the included NRSs, none of these were conducted before 2009. Eight NRSs provided outcome measurements indicating limb salvage rate (ie, free of above-ankle amputation) or free from major amputation rate. One NRS presented only the free from amputation rate and did not indicate major or minor amputation rate. Seven studies recorded the wound healing rate, and 1 study recorded the wound unhealing rate. The NRS by Rashid et al^28^ was excluded because their results provided subgroup data only, which could not be analyzed. All included trial patients were in either Rutherford Class 5 or 6 and Fontaine Stage 4.^29,30^ The key characteristics of patients included in all studies are shown in Table 2. Mean patient age was >67 years old and most patients were male. In addition, the majority of patients (at least 64%) had diabetes mellitus (DM), and 3 studies recruited only DM patients.^21,25,26^ We did not perform further subgroup analysis because only Varela et al^19^ had described the subgroup of IR with collateral vessels.

**TABLE 1:**
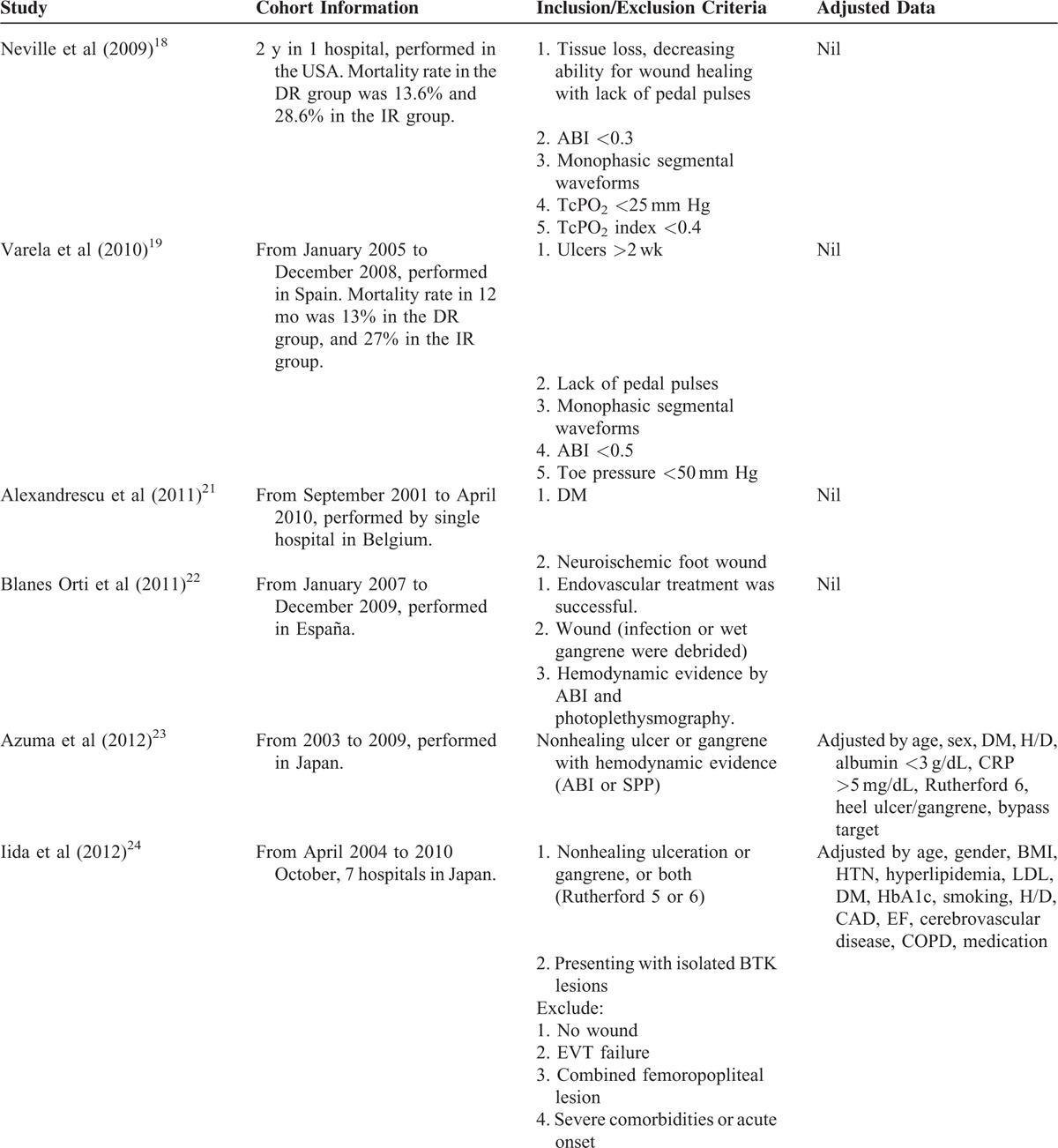
Studies Included in the Systemic Review of the Effects of Angiosome Model Revascularization Treatment for Patients With Low Limb Ischemia

**TABLE 1 (Continued):**
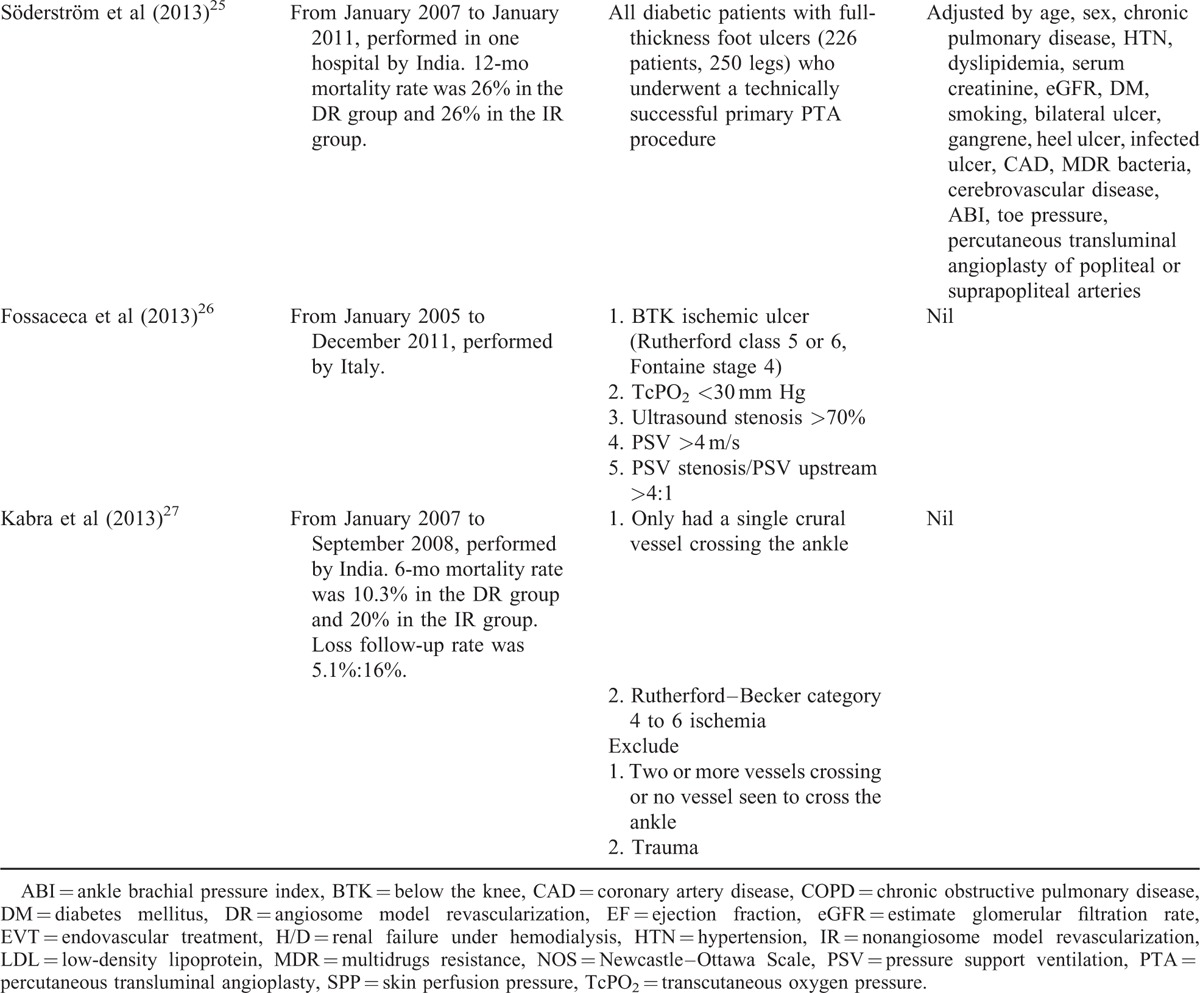
Studies Included in the Systemic Review of the Effects of Angiosome Model Revascularization Treatment for Patients With Low Limb Ischemia

**TABLE 2:**
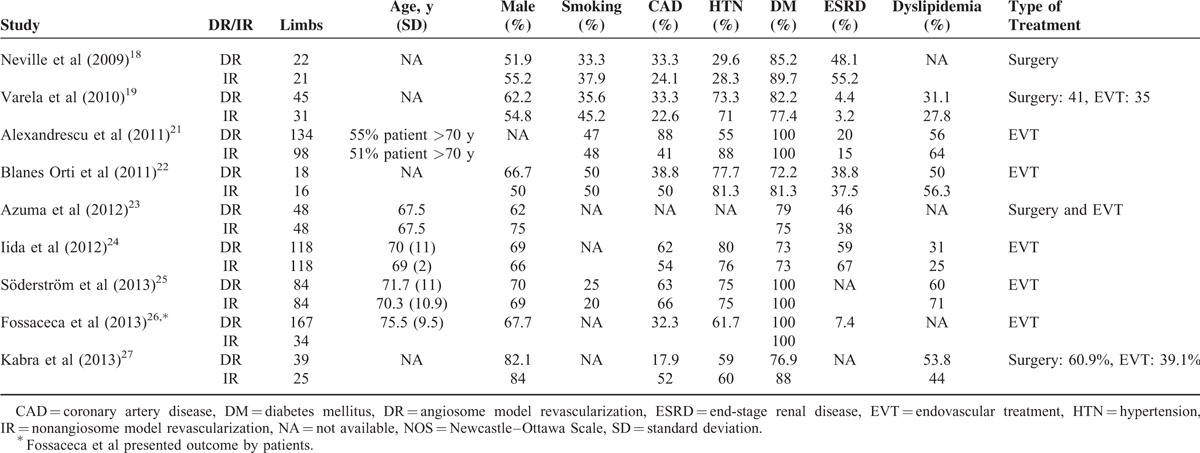
The Patient Characteristics of the Included Studies

### Quality Assessment

The risks of bias for all NRSs are shown in Table 3. The NOS of all trials ranged from 5 points to 9 points. Five NRSs had a score of >8 points.

**TABLE 3:**
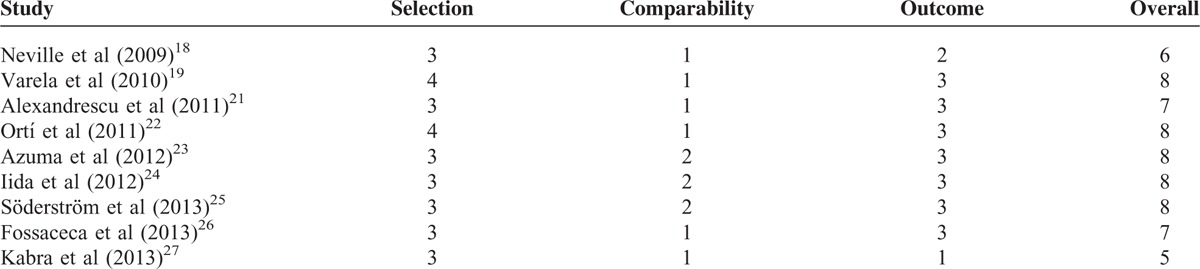
The Newcastle–Ottawa Scale of Included Studies

### Meta-Analysis of Limb Salvage and Wound Healing

Reported outcomes in primary studies were shown in Table S1, http://links.lww.com/MD/A394, Table S2, http://links.lww.com/MD/A394, and Table S3, http://links.lww.com/MD/A394. Although 9 NRSs reported a limb salvage rate,^18,19,21–27^ and 8 NRSs reported the wound healing rate, the follow-up periods were different in individual study.^18,19,21–23,25–27^ To include more studies into our meta-analysis, we used time to amputation and time to wound healing as our primary endpoints. In addition, we could not find adequate information with regard to the data of loss-to-follow-up in primary studies. However, we analyzed 12-month amputation rate and 12-month wound healing rate using available information. A total of 719 limbs were treated with DR, and 493 limbs were treated with IR. Five studies included total endovascular treatment (EVT), another study included traditional bypass surgery, and 3 studies included both surgical and EVT interventions.

Fossaceca et al^26^ recorded the limbs of the DR and IR groups (247 limbs vs 52 limbs), but only presented outcomes by counting individual patients (167 patients vs 34 patients). Therefore, we calculated the data in our meta-analysis according to the number of patients.

### Time to Amputation

**FIGURE 2:**
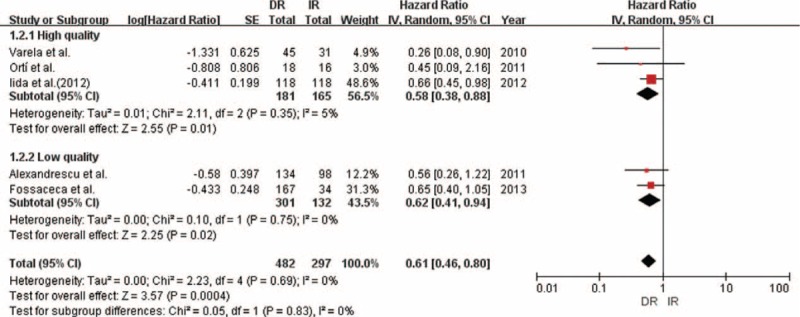
Forest plot comparing time to amputation, stratified by study quality.

**FIGURE 3:**
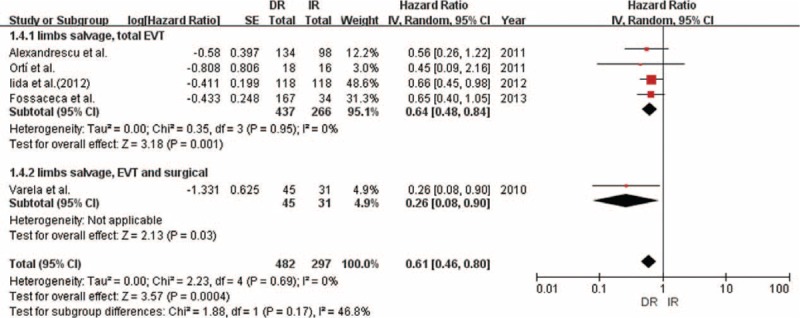
Forest plot comparing time to amputation, stratified by operation method.

Overall, we obtained 5 studies that reported 779 limbs. DR significantly improved time to amputation compared with IR (HR 0.61; 95% CI = 0.46–0.80; *P* < 0.001; Table 4; Figures 2 and 3). Little evidence of heterogeneity between studies was obtained (*P* = 0.69, *I*^2^ = 0%). According to study quality and intervention methods, subgroup analysis was performed, which showed little evidence of interaction (Table 4).

**TABLE 4:**
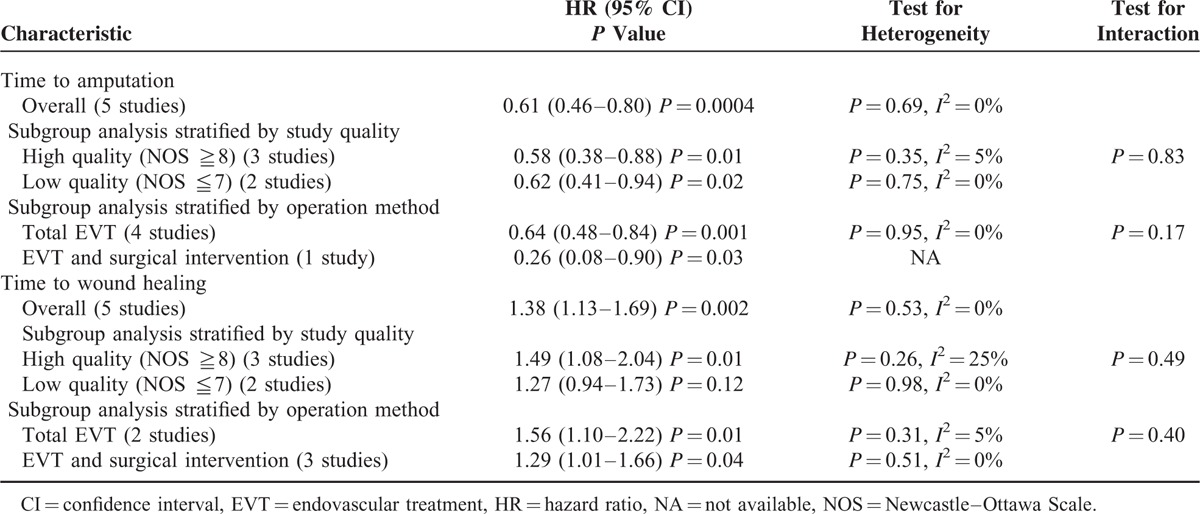
Hazard Ratios for Time to Amputation and Time to Wound Healing for Patients Receiving Angiosome Model Target Revascularization Compared With Nonangiosome Group According to Meta-Analysis and Subgroup Analysis of All Trials

We analyzed 12-month amputation rate using 4 available studies. Our result showed the DR group significantly reduced 12-momth amputation rate compared with IR group (relative risk ratio [RR] 0.65; 95% CI = 0.54–0.79; *P* < 0.001; Figure S1, http://links.lww.com/MD/A394).

### Time to Wound Healing

**FIGURE 4:**
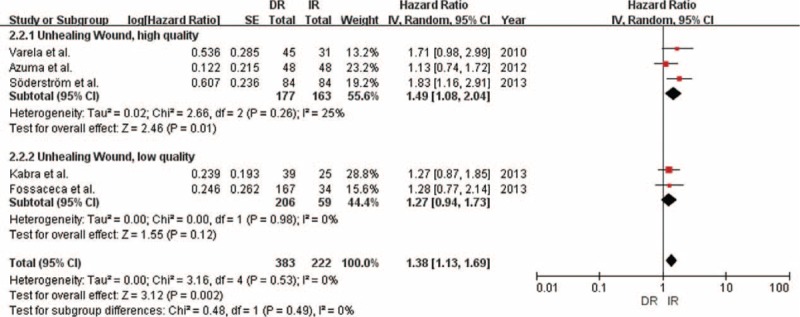
Forest plot comparing time to wound healing, stratified by study quality.

**FIGURE 5:**
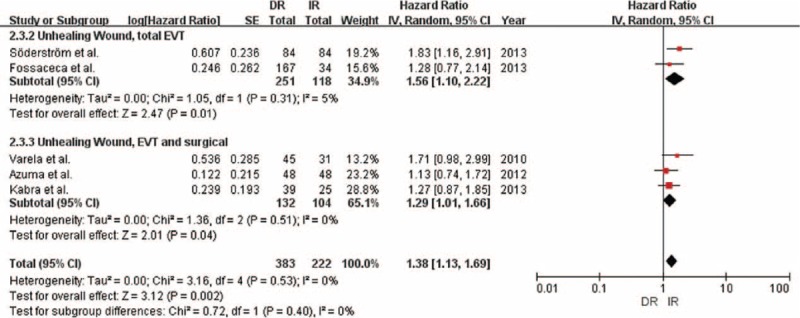
Forest plot comparing time to wound healing, stratified by operation method.

We obtained similar results for time to wound healing in the 5 studies with 605 limbs analyzed in which DR exerted a statistically significant effect (HR 1.38; 95% CI = 1.13–1.69; *P* = 0.002; Table 4; Figures 4 and 5). We found little heterogeneity between studies (*P* = 0.53; *I*^2^ = 0%). Subgroup analyses according to study quality and intervention methods showed little evidence of interaction (Table 4).

We analyzed 12-month wound healing rate using 4 available studies. Our result showed the DR group significantly improved 12-momth wound healing compared with IR group (RR 1.45; 95% CI = 1.26–1.66; *P* < 0.001; Figure S2, http://links.lww.com/MD/A394).

### Mortality Rate

Mortality rate was only reported in 4 studies. Neville et al^18^ described a mortality rate of 13.6% over 100 days in the DR group and of 28.6% in the IR group. Kabra et al^27^ reported a mortality rate of 10.3% over 6 months in the DR group and of 20.0% in the IR group. Varela et al^19^ showed 13% mortality rate in the DR group and 27% in the IR over 12 months, as well as a *P* value of 0.17. Söderström et al^25^ showed 1-year survival rates of 74% in both the DR and IR groups (*P* = 0.65).

### Publication Bias

**FIGURE 6:**
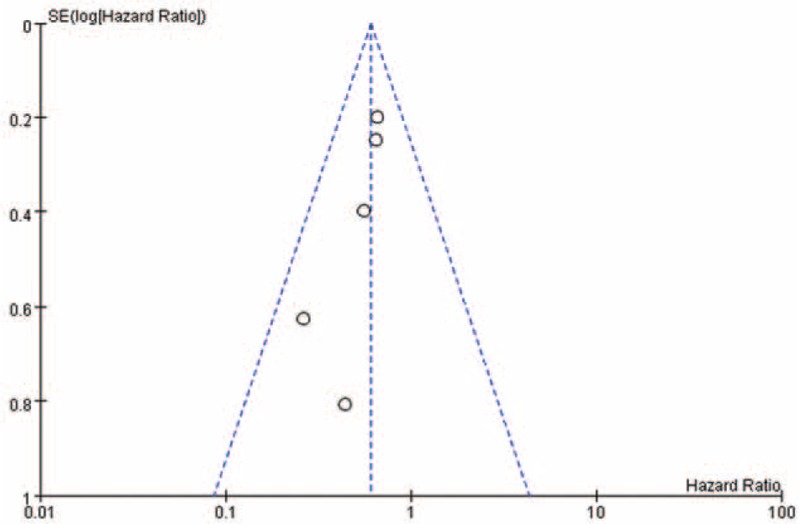
Funnel plot of studies comparing time to amputation.

**FIGURE 7:**
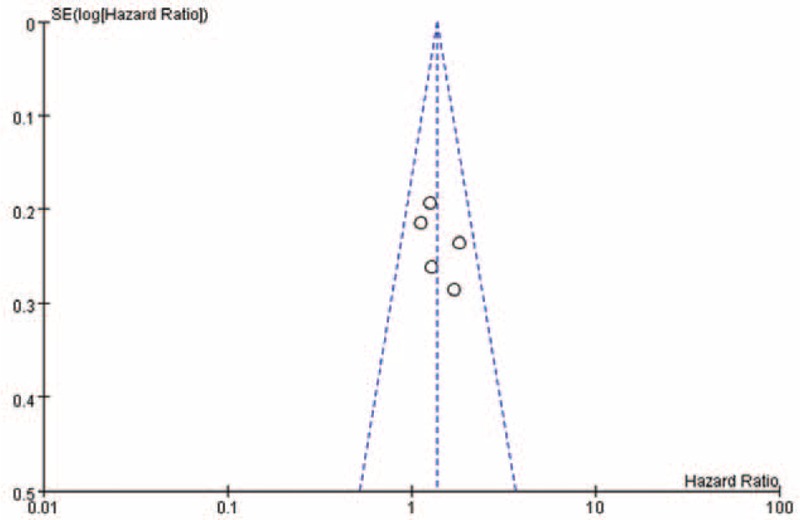
Funnel plot of studies comparing time to wound healing.

Publication bias was analyzed using a funnel plot, which was symmetrical (Figures 6 and 7).

## DISCUSSION

Our meta-analysis provided evidence that DR significantly improves time to amputation and time to wound healing for CLI patients. However, insufficient information was available to conduct a meta-analysis on mortality. The angiosome concept was constructed based on the anatomy of blood circulation, which demonstrates that superior blood supply can improve tissue growth and wound healing.

Three studies performed matching in their data analysis (Azuma et al, Iida et al, and Söderström et al).^23–25^ The result of Iida et al showed that DR significantly decreased the amputation rate (HR 0.66; 95% CI = 0.45–0.98; *P* = 0.04). Azuma et al and Söderström et al were also comparing the outcome of time to wound healing. Söderström et al supported that DR group significantly increased wound healing rate (*P* < 0.001). However, Azuma et al showed no difference for wound healing between 2 groups (*P* = 0.185).

Varela et al confirmed that DR model treatment improved the wound healing rate 12 months following intervention (92% vs 73%; *P* < 0.01) and limb salvage rate 24 months following intervention (93% vs 72%; *P* = 0.02) for CLI patients. Varela et al further revealed that distal peroneal arterial connections (collateral vessels) and the patent pedal arch played a significant role in wound healing and limb salvagability in CLI patients who were treated without using the DR model.^19^ This suggested that the possible cause of IR treatment failure resulted from inadequate vascular connections between the revascularized arteries and the ischemic region. Therefore, a patent pedal arch or peroneal distal branches that restore blood flow to the ischemic area through collateral vessels might show a similar result in limb salvagability and wound healing as that obtained through the specific source arteries.^19^

Although current AHA/ACC guidelines suggest open bypass still the preferred operation for patients who would live for >2 years, traditional surgery and EVT have been compared in several studies.^31–35^ One meta-analysis performed by Romiti et al compared surgical and EVT interventions and demonstrated no difference in the limb salvage rate (endovascular, 82.4% ± 3.4%; surgery, 82.3% ± 3%).^36^ Advantages of EVT intervention include less surgical trauma, a smaller wound, fewer local complications, and shorter hospital stays.^31,32^ However, subgroup analysis for comparisons between EVT intervention and surgical intervention could not be assessed in this study.

DM has been recognized as a critical predicting factor for wound healing. Failure of ulcers to heal in the feet of diabetic patients might have resulted from poor vascular connections between angiosomes, which provided inadequate blood perfusion to the ischemic areas. In addition, TASC II guidelines indicate that the amputation rate was 5 to 10 times higher in diabetic patients than in nondiabetic patients because blood flow to the microvascular beds appears to be reduced in the feet of diabetic patients.^2^ Furthermore, diabetic patients have an impaired host defense system against infections.^37,38^ Azuma et al^23^ stated that diabetes is one of the risk factors in prolonged tissue healing time. Iida et al showed that higher hemoglobin A_1c_ levels were a significant predictor of major amputation in a DR group. They postulated that the increased risk is most likely attributable to poor periprocedural blood glycemic control rather than to the presence of DM during the postoperative period.^24^ Three studies compared DR in diabetic patients.^21,25,26^ However, our study compared the outcomes of these 3 studies with the others, which had a distinct percentage of diabetic patients (>64%), and minimal heterogeneity was identified (*P* = 0.40; *I*^2^ = 0%). No other studies comparing diabetic and nondiabetic patients have been published. DR might have beneficial effects for wound healing in diabetic patients; however, the effects on nondiabetic patients require further investigation.

Azuma et al^23^ proved that DR treatment could significantly shorten the time needed for wound healing in the entire study cohort, and that it improved limb salvage rate in end-stage renal disease (ESRD) patients (*P* < 0.01). However, after propensity-score matching, the differences between limb salvage rate and wound healing rate in the DR and IR groups were lost. Azuma el al concluded that the angiosome concept might be unimportant in the field of bypass surgery, unlike EVT intervention. However, our analysis revealed that the DR model concept could be consistently applied to all patients, regardless of surgical or EVT intervention.

ESRD was a crucial risk factor for wound healing and limb salvagability,^23^ and several studies have reported that patients with ESRD have higher amputation rates.^39–41^ Johnson et al^39^ postulated that healing problems account for higher amputation rates rather than graft thrombosis. Thus, some studies have recommended that bypass surgery should be performed on carefully selected ESRD patients because of potential negative outcomes.^39,42,43^ However, no standard exists for selected ESRD patients.^44^ Some studies have reported that hypoalbuminemia, which might result from inflammation instead of malnutrition,^45,46^ detrimentally related to the life prognosis of ESRD patients.^23,31,41^ Azuma et al separated their patients into 3 groups: non-ERSD, ESRD without severely low albuminemia, and ESRD with severely low albuminemia (<3.0 g/dL). The wound healing rate of the ESRD in the low albuminemia group was significantly worse than in the other groups (*P* < 0.01). Their subgroup analysis demonstrated that DR significantly improved wound healing rate both in non-ESRD patients and in ESRD patients as compared with IR. However, comparing DR and IR in all patients showed no beneficial effect in their study (*P* = 0.185). They concluded that ESRD and the level of serum albumin were more critical than the angiosome concept.^23^

Cilostazol was typically used in PAOD patients as an antiplatelet drug.^47^ The improvement of microvascular circulation has been reported as one of the clinical benefits of cilostazol.^48^ Iida et al formed 2 groups and recorded the outcome of skin perfusion pressure (in mm Hg) on the time before and after surgery.^24^ Skin perfusion pressure was similar to that before intervention (1.6 ± 0.9 with cilostazol therapy vs 1.6 ± 0.8 without, *P* = 0.91), and it was statistically higher after EVT in the cilostazol-treated group than in the noncilostazol-treated group (51 ± 19 vs 45 ± 19, *P* = 0.04).^24^ Therefore, cilostazol might help to improve microcirculation; however, further evidence of amputation prevention or improvement in wound healing is necessary.

In this study, we performed throughout literature searches. For evaluation of time-to-event outcomes, we utilized HR as the metric of the summary effect size. The calculated HR was the relative hazard of an event occurring in the DR group compared with that of the IR group. From our result, it suggested that patients who received DR had decreasing risk of limb amputation and wound unhealing by 0.61 (95% CI = 0.46–0.80) and 1.38 (95% CI = 1.13–1.69) in the PAOD patients compared with patients received IR interventions. However, our study has several limitations. First, all included studies were retrospective comparative studies. Angiosome concept was delicate. It was trivial to approach the target feeding artery for the ischemia area. Therefore, there were too many operation methods for vascular access that all the retrospective studies could not have a very specific and appropriate design. Second, another source of bias might come from the confounding bias because of patients’ condition. Only Azuma et al, Iida et al, and Söderström et al performed matching in their analyses. The bias existed among these studies resulting from the differences of the selection and grouping criteria for these patients. The mortality rate of the patients treated with IR was higher than those treated with DR might imply that the patients in the IR had more comorbidities than those in the DR group. Third, no consensus was obtained in describing wound conditions in the included studies. The Rutherford and Fontaine stage was used in only 4 studies, but they only recorded stages. The locations, numbers, infection status, and surgical debridement procedures of these wounds were not recorded in numerous studies, which were major confounders in our analysis. The wound healing rate and the treatment strategy for a gangrenous wound would be much different than that of a superficial ulcer. None of the studies reported postoperative wound care programs. Infections, antibiotic treatment, and debridement surgery are all additional concerns in the included studies. Fourth, the detail description for the vascular lesions were absent. Future randomized study should report the detail of these vascular lesions, such as length, location, stenotic status, collateral vessel of the lesions as well as the status of the pedal arch. It is possible that the patients in the DR group exhibited superior vascular quality and that the target vessel was more easily approached, whereas patients in the IR group might have had either total occlusion or vessels that were small in diameter and had degenerated. Thus, the 2 groups were at distinct stages.

In conclusion, the angiosome model of revascularization was beneficial for patients with critical lower limb ischemia when considering limb salvagability and wound healing. Nonetheless, randomized controlled studies are necessary to confirm our results. Increasing the limb salvage rate is anticipated to improve daily activity and could prolong the survival of patients. Thus, a broad prospective study should be conducted to confirm the effect of the angiosome model concept. All the following characteristics should be recorded in detail, including the wound condition and location with standard recording system, the treatment of the wound (debridement, antibiotics, and the dressing of wound), the detail description of the stenotic status, the collateral vessels, and the condition of the pedal arch. Collateral vessels should be defined more carefully because some patients, especially diabetic patients, are collateral artery-dominant blood supply. Crucial confounding factors, such as DM, ESRD, serum albumin levels, and medications, should also be reported and analyzed.
